# Treatment of Sciatica

**Published:** 1856-06

**Authors:** Peyton Blakiston


### TREATMENT OF SCIATICA.

BY PEYTON BLAKISTON, F. R. S.

Dr. Blakiston, has pursued the following treatment for twenty years with considerable success. He first saw it adopted in Paris in 1833: A blister, about the size of a crown-piece, is placed over the chief seat of pain, which is usually the flattened part of the buttock. After it has risen well, and the cuticle has been thoroughly removed, the raw surface is sprinkled with a powder, consisting of one grain of acetate of morphia on an average, and a little white sugar. This dressing is repeated for six successive days, the surface of the blister being kept in a raw state, if requisite, by cantharides or savine cerate, or else by Albuspeyeres' plaster. This suffices for a very mild case; but in severe cases of old standing, the pain will now be found to have left it original seat, and to have seized on the knee of the affected side. The same treatment is then applied to the ham; and after six dressings, the pain will have generally disappeared, and the patient will rapidly recover. By this mode of treatment, eighty-three cases of uncomplicated sciatica have been cured, without failure having come to the knowledge of the writer. This number might have been greatly augmented had it included the results arrived at by such of his friends and former pupils as had employed it at his suggestion, and which have been no less successful than those which occurred in his practice; but he is desirous of recording such only as have come under his own immediate notice, and for the accuracy of which he consequently can hold himself responsible. In the great majority of these cases no other drug was administered; but in a few, some laxative medicine or injection was given to remove constipation. In two or three cases, there was a tendency to double sciatica, and then the pain passed from the sciatic region first treated to that of the opposite side, and from thence down to the knee of this last side, but never attacked the knee of the side first affected. It is right to mention, that in hospital practice three cases were placed under the writer's care, which he considered more than doubtful, and they were therefore treated under protest, so to speak. They all turned out cases of hip disease, and therefore they are not included in those above enumerated. The difference in the sensation felt by the patients on the first application of the morphia was remarkable; and without any attempt to generalize, it may be stated that a close connexion was observed between the sensations felt and the previous state of health. Thus the effect produced on three persons in robust health—a blacksmith, a gamekeeper, and a lady—was most intense; an extraordinary thrilling was felt over the whole body, particularly at the extremities, with great nausea, and a tendency to faint. The lady vomited incessantly for twelve hours, so that it was found advisable to reduce the quantity of morphia in the powders to half a grain. On the other hand, a gentleman, who had been much reduced by overwork and long suffering, felt no effect whatever from the application of the powders, and yet he recovered in an equally short time with the others. A lady, also, who had been taking considerable doses of opium, hardly felt the application of morphia until it was increased to two grains; but this case has been excluded, because, although the sciatica was removed by the treatment, there remained an uncurable disease, which eventually destroyed her. One lady, aged 26, in whom the disease was not of long standing, obstinately refused to have a second powder applied; but happily the one application sufficed to effect a cure. In six cases the disease recurred after an interval of from five to eighteen months; and in two of these it recurred twice; but each attack was less severe than the one which preceded it and yielded readily to the same treatment. It is possible, however, that relapses might have more frequently taken place without having come under the notice of the writer; but he thinks this cannot have happened very often. Some other forms of neuralgia were also benefitted by this mode of treatment. Thus a very distressing case of neuralgia of the scalp yielded at once; and shooting pains, which frequently accompany cancer of the stomach, were sometimes much relieved by it.—*Med. Times and Gaz.*, *in Dublin Med. Press.*

